# HerMeS: a registry-based evaluation of the HERCULES criteria for identifying nonrelapsing SPMS

**DOI:** 10.1007/s00415-026-13716-1

**Published:** 2026-03-09

**Authors:** Tommaso Guerra, Giuseppe Lucisano, Maria A. Rocca, Eleonora Cocco, Francesco Patti, Giovanna De Luca, Emilio Portaccio, Matteo Foschi, Diana Ferraro, Carlo Pozzilli, Silvia Romano, Pietro Annovazzi, Vincenzo Brescia Morra, Carla Tortorella, Roberta Fantozzi, Paola Perini, Maria Gabriella Coniglio, Giuseppe Salemi, Matilde Inglese, Giorgia Teresa Maniscalco, Valentina Liliana Adriana Maria Torri Clerici, Alessandra Lugaresi, Giacomo Lus, Elena Colombo, Franco Granella, Paola Cavalla, Alessia Di Sapio, Antonella Conte, Raffaella Cerqua, Rocco Totaro, Francesca Caputo, Damiano Paolicelli, Alberto Farina, Margaret Mary Mondino, Daniele Santo Todaro, Nunzio Olivieri, Maria Pia Amato, Massimo Filippi, Maria Trojano, Pietro Iaffaldano

**Affiliations:** 1 Department of Translational Biomedicine and Neurosciences, DiBraiN, University of Bari “Aldo Moro”, Multiple Sclerosis Center, Policlinico of Bari, Bari, Italy; 2 Center for Outcomes Research and Clinical Epidemiology, Pescara, Italy; 3 Division of Neuroscience, Neuroimaging Research Unit, IRCCS San Raffaele Scientific Institute, Milan, Italy; 4 Vita-Salute San Raffaele University, Milan, Italy; 5 Neurology Unit, IRCCS San Raffaele Scientific Institute, Milan, Italy; 6 Department of Medical Science and Public Health, University of Cagliari, Cagliari, Italy; 7 Dipartimento di Scienze Mediche e Chirurgiche e Tecnologie Avanzate, GF Ingrassia, Università di Catania, Catania, Italy; 8 UOS Sclerosi Multipla, AOU Policlinico “G Rodolico-San Marco”, Università di Catania, Catania, Italy; 9 Centro Sclerosi Multipla, Clinica Neurologica, Policlinico SS Annunziata, Università “G. d’Annunzio”, Chieti-Pescara, Italy; 10 Department of NEUROFARBA, University of Florence, Florence, Italy; 11 Department of Neuroscience, Multiple Sclerosis Center-Neurology Unit, S. Maria Delle Croci Hospital of Ravenna, AUSL Romagna, Ravenna, Italy; 12 Department of Biotechnological and Applied Clinical Sciences, University of L’Aquila, L’Aquila, Italy; 13 Department of Neurosciences, Ospedale Civile di Baggiovara, Azienda Ospedaliero-Universitaria di Modena, Modena, Italy; 14 Department of Human Neurosciences, Sapienza University of Roma, Rome, Italy; 15 Department of Neurosciences, Mental Health and Sensory Organs (NESMOS), Sant’Andrea Hospital, Sapienza University of Rome, Rome, Italy; 16 Neuroimmunology Unit and Multiple Sclerosis Center, ASST Della Valle Olona, Hospital of Gallarate, Gallarate, VA Italy; 17 Department of Neurosciences, Reproductive and Odontostomatological Sciences, Multiple Sclerosis Clinical Care and Research Center, University of Naples Federico II, Naples, Italy; 18 Dipartimento di Neuroscienze, Ospedale San Camillo-Forlanini, Rome, Italy; 19 IRCCS Neuromed, Pozzilli, Italy; 20 Multiple Sclerosis Centre of the Veneto Region (CeSMuV), Neurology Clinic, University Hospital of Padua, Padua, Italy; 21 Center for Multiple Sclerosis, ASM P.O. “Madonna Delle Grazie”, Matera, Italy; 22 Department of Biomedicine, Neurosciences and Advanced Diagnostics, University of Palermo, Palermo, Italy; 23 IRCCS Ospedale Policlinico San Martino, Genoa, Italy; 24 Dipartimento di Neuroscienze, Riabilitazione, Oftalmologia, Genetica e Scienze Materno Infantili (DINOGMI), Universita’ di Genova, Genova, Italy; 25 Dipartimento di Neurologia e Stroke Unit, Centro Regionale Sclerosi Multipla, Ospedale “A. Cardarelli”, Naples, Italy; 26 Fondazione IRCCS Istituto Neurologico Carlo Besta, Milan, Italy; 27 IRCCS Istituto Delle Scienze Neurologiche di Bologna, Bologna, Italy; 28 Dipartimento di Scienze Biomediche e Neuromotorie, Università di Bologna, Bologna, Italy; 29 Department of Advanced Medical and Surgical Sciences, University of Campania “Luigi Vanvitelli”, Naples, Italy; 30 IRCCS Mondino Foundation, Pavia, Italy; 31 Department of Medicine and Surgery, Unit of Neurosciences, University of Parma, Parma, Italy; 32 Department of Neuroscience “Rita Levi Montalcini”Multiple Sclerosis Center, City of Health and Science University Hospital of Torino, University of Turin, Turin, Italy; 33 Department of Neurology, Regional Referral Multiple Sclerosis Center, University Hospital San Luigi Gonzaga, Orbassano, Turin, Italy; 34 Dept. of Experimental and Clinical Medicine, Neurological Clinic, AOU Delle Marche, Torrette, Italy; 35 Centro Malattie Demielinizzanti, Clinica Neurologica, Ospedale San Salvatore, L’Aquila, Italy; 36 Medical Department, Sanofi, Milan, Italy; 37 IRCCS Don Carlo Gnocchi, Florence, Italy; 38 University of Bari “Aldo Moro”, Bari, Italy

**Keywords:** Multiple sclerosis, Secondary progression, Nonrelapsing SPMS, Disease registry, Data-driven algorithm

## Abstract

**Background:**

Secondary progressive multiple sclerosis (SPMS) encompasses heterogeneous phenotypes that may or may not exhibit disease activity.

**Objectives:**

To characterize a cohort of non-relapsing SPMS (nrSPMS) identified through a data-driven definition adapted from the HERCULES trial criteria, compared with neurologist-defined (ND) SPMS.

**Methods:**

Relapsing MS patients were retrospectively extracted from the Italian Multiple Sclerosis Register (RISM). ND was defined according to clinical criteria for SPMS and the HERCULES cohort adapting the trial inclusion criteria. Progression independent from relapse activity (PIRA) and relapse-associated worsening (RAW) events were assessed. Diagnostic performances of SPMS definitions were evaluated.

**Results:**

Among 20,306 patients, 866 (4.26%) were classified as SPMS by ND and 1603 (7.89%) by HERCULES criteria. The HERCULES group included older patients, less likely to relapse post-conversion. PIRA occurred in 88.8% of ND and 88.5% of HERCULES-defined cases, with a shorter median time to first PIRA in the latter. The HERCULES definition showed high specificity (93.4%) and low sensitivity (37.6%).

**Conclusions:**

HERCULES-adapted criteria identified a subgroup of 7.9% nrSPMS patients in the real-world cohort of RISM, characterized by fewer post-conversion relapses and earlier PIRA onset. These findings reinforce the value of applying standardized algorithmic definitions of SPMS in registry-based studies.

**Supplementary Information:**

The online version contains supplementary material available at 10.1007/s00415-026-13716-1.

## Introduction

The neuropathological continuum of multiple sclerosis (MS), sustained by inflammatory demyelination and neurodegeneration, has been delineated by major studies over the last few years [1–3]. The presence of progression independent of relapse activity (PIRA) since the disease onset demonstrates that progression phenomena characterize MS throughout its course, challenging the classic clinical distinction in phenotypes. [4] However, this subdivision is widely applied and underpins prescription and reimbursement criteria for disease-modifying therapies (DMTs). [5, 6] Despite its clinical meaning, the definition of secondary progressive multiple sclerosis (SPMS) remains challenging because of characteristics that overlap between the relapsing and progressive phases: even when PIRA accounts for the majority of the disability accrual in SPMS, representing more than 80% of confirmed disability accumulation events, relapses may still occur. [7] Therefore, SPMS patients have been further categorized as "active" or "non-active" based on the presence of relapses or radiological activity. [8] A previous analysis conducted by the Italian Multiple Sclerosis and Related Disorder Register (RISM) group demonstrated the superior ability to capture the SP transition of two different data-driven SPMS definitions, based on a version of Lorscheider’s algorithm and the EXPAND trial inclusion criteria, compared to the neurologist’s definition (ND). [9] Recent clinical trials in MS including nonrelapsing SPMS (nrSPMS) have prompted discussion over implications for clinical practice. [10, 11] The HERCULES trial, investigating the efficacy and safety outcomes of tolebrutinib, a Bruton tyrosine kinase inhibitor, targeted a nrSPMS population to assess the effect on compartmentalized inflammation [12, 13], as a result of smoldering pathological processes [14, 15]. The therapeutic and diagnostic challenges are therefore intertwined. The present study had three objectives. First, we aimed to describe the clinical characteristics of SPMS populations identified according to ND and an adapted definition of the HERCULES criteria, using data from the RISM, a large and validated database representative of the Italian MS population. In this context, we investigated the incidence of PIRA events. Second, we aimed to assess the diagnostic performance of the adapted HERCULES definition, examining its ability to capture an nrSPMS phenotype. Third, we aimed to compare the timing of SPMS identification according to ND and adapted HERCULES criteria, to explore potential differences in diagnostic timing between definitions.

## Material and methods

### Study approval and patient consents

The analysis was conducted using data from RISM, previously authorized by the Ethics Committee of the Azienda Ospedaliero-Universitaria Policlinico of Bari (REGISTRO SM001) and by the local ethics committees.

### Study population, outcome definition and SPMS definitions

This was a retrospective observational cohort study using data from RISM. Data extraction was executed in January 2024. Patient data retrieved from the RISM included demographic, clinical, magnetic resonance imaging (MRI), and treatment-related characteristics. The selection criteria for patients included relapsing-onset MS patients (RMS) with age at onset of at least 18 years, a first visit within one year of disease onset, and at least three Expanded Disability Status Scale (EDSS) evaluations over time.

As reported in previous studies [4, 15], the confirmed disability accrual (CDA) was defined as a confirmed 6-month disability increase from study baseline, measured by EDSS (increase ≥ 1.5 points with baseline EDSS = 0; ≥ 1.0 point with baseline EDSS > 1.0, and < 5.5; ≥ 0.5 point with baseline EDSS > 6.0). Date of CDA was assigned at the first EDSS when the disability increase was registered. PIRA, the primary outcome investigated, was defined as a CDA event occurring more than 90 days after, and more than 30 days before, the onset of a relapse. Relapse-associated worsening (RAW) was defined as a CDA event in which the initial disability worsening from study baseline occurred within 90 days after, or within 30 days before, the onset of a relapse. [4].

The following different definitions have been used to identify SPMS patients included in the RISM cohort:

- Neurologist Definition (ND): as reported in our previous study [9], SPMS identification was based on the treating neurologist’s clinical judgment consistent with the Lublin criteria. [17] The SPMS conversion date was derived from clinical records entered in the RISM.
- nrSPMS—HERCULES adapted definition criteria: adapted from the HERCULES trial SPMS criteria [12], a data-driven definition requiring a relapsing onset, age ≥ 18 years, EDSS between 3.0 and 6.5 at the time of SP conversion, CDA (24-week or later confirmed disability increase from study baseline) without clinical relapses for at least 24 months before SP conversion

For the primary objective, the variables assessed included all available clinical and demographic characteristics recorded in the RISM before and after SPMS labeling. Geographical sub-analyses were performed by describing the distribution of SPMS definitions across Northern, Central, and Southern Italian regions within the RISM network. Time to SPMS labeling was assessed according to ND and adapted HERCULES criteria and was defined as the interval between disease onset and SPMS diagnosis under each definition.

### Statistical analysis

For descriptive results, continuous data are presented as mean and standard deviation (SD) for approximately normally distributed variables, and as median and interquartile range (IQR) for skewed distributions or variables with outliers. Baseline clinical characteristics were described and stratified according to the two cohorts defined by the different SPMS definitions. Given the descriptive nature of the study, and because ND- and adapted HERCULES-defined SPMS cohorts are not mutually exclusive and may overlap at the individual level, no formal hypothesis testing was performed.

We considered different measures to evaluate the diagnostic performance and the agreement of the data-driven definitions of SPMS according to HERCULES compared to the ND, used as the reference: calibration (agreement between predicted and observed outcomes), discrimination (ability to distinguish SPMS from non-SPMS), and overall model fit. In the absence of a universally accepted gold standard for SPMS diagnosis, we decided to apply neurologist-defined (ND) SPMS as a pragmatic reference classification reflecting real-world clinical labeling [9].

The overall calibration of the data-driven definitions was evaluated by the Calibration Slope test, with* p* > 0.05 indicating good calibration. Discrimination metrics were derived from standard classification indices: true positive (TP), false negative (FN), false positive (FP), true negative (TN), specificity, sensitivity, positive predictive value (PPV), negative predictive value (NPV), and accuracy. Model fit was evaluated using Akaike Information Criterion (AIC).

We included Wilson confidence intervals for performance indices.

Time to SPMS identification according to ND and adapted HERCULES criteria was evaluated using time-to-event methods applied to the entire eligible cohort. Cumulative incidence curves were estimated using Kaplan–Meier methods to illustrate the timing of SPMS identification according to each definition, as well as the occurrence of PIRA over follow-up. Exploratory Cox regression models were used to assess associations between baseline clinical factors (at the first visit recorded in RISM) and SPMS identification according to ND and adapted HERCULES criteria. In addition, we performed a descriptive subgroup analysis based on the geographical distribution of MS centers where patients are followed,

Analyses were performed using R, version 4.3.3 (R Foundation for Statistical Computing).

## Results

**Fig. 1:**
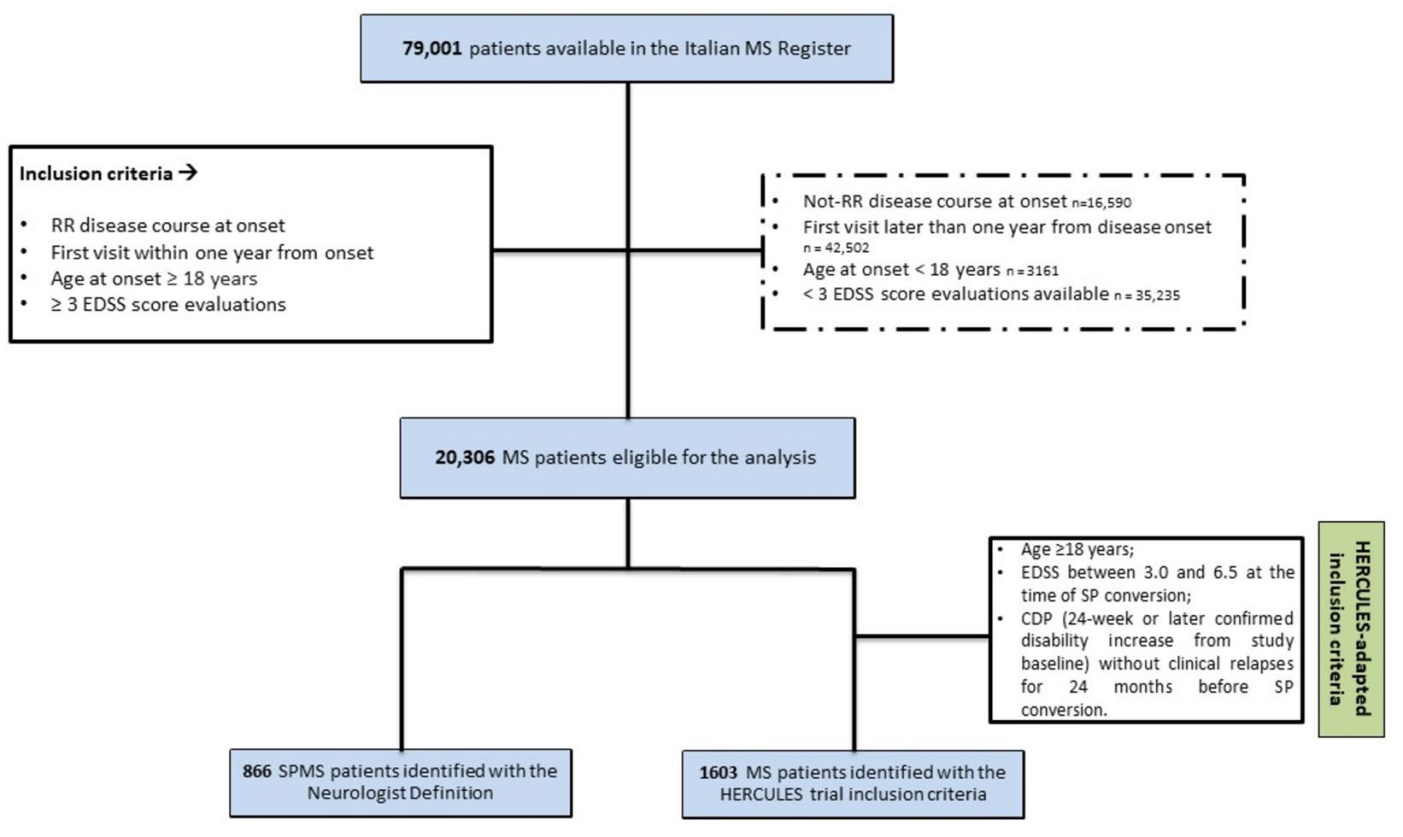
Flowchart of patient selection

Clinical records from a total of 79,001 MS patients were accessible within the RISM at the time the dataset was extracted. After applying the inclusion criteria, a cohort of eligible 20,306 RMS patients from 125 MS centers of the RISM network was identified. Among them, 866 (4.26%) were classified as SPMS according to ND and 1603 (7.89%) according to the HERCULES definition. Figure 1 provides a schematic overview of the patient selection procedure. Clinical and demographic characteristics at SPMS conversion are reported in Table 1. For descriptive purposes, Table 1 also reports clinical characteristics of patients who did not convert to SPMS during follow-up in the total eligible RMS cohort. In the eligible cohort, median follow-up duration was 16.9 years (IQR 11.0–22.8) for ND-defined patients and 13.6 years (IQR 8.3–19.0) for HERCULES-defined patients. We identified at least one PIRA event throughout the follow-up in 8089 (39.8%) patients.

**Table 1:** Baseline clinical and demographic characteristics

	RMS patients without SPMS conversion	ND	HERCULES-adapted definition
Number of patients identified	18,163	866	1603
Follow-up, median (IQR), years	6.20 (2.80–11.40)	16.90 (11.00–22.80)	13.60 (8.30–19.00)
Time from first visit to SPMS conversion, median (IQR) years	-	7.08 (3.61–12.34)	7.00 (3.50–12.15)
Age at the analysis, median (IQR) years	40.34 (32.73–48.79)	44.45 (37.40–51.70)	46.60 (39.20–53.40)
Number (%) of subjects with age ≤ 50	14,213 (78.25)	609 (70.32)	989 (61.70)
Number (%) of subjects with age > 50	3950 (21.75)	257 (29.68)	614 (38.30)
Male sex, *n* (%)	5925 (32.62)	334 (38.57)	573 (35.75)
EDSS, median (min–max)	1.50 (1.00–2.00)	4.50 (3.00–5.50)	4.00 (3.00–4.50)
EDSS categories			
0.0	3038 (16.73)	29 (3.35)	0 (0.0)
1.0–1.5	8162 (44.94)	44 (5.08)	0 (0.0)
2.0–2.5	4171 (22.96)	100 (11.55)	0 (0.0)
3.0–3.5	1156 (6.36)	127 (14.67)	785 (48.97)
4.0–4.5	736 (4.05)	209 (24.13)	427 (26.64)
5.0–5.5	285 (1.57)	142 (16.40)	178 (11.10)
6.0–6.5	427 (2.35)	169 (19.52)	212 (13.23)
≥ 7.0	188 (1.04)	29 (3.35)	0 (0.0)
Patients with relapses in the previous year before SPMS, *n* (%)	1753 (9.65)	319 (36.84)	0 (0.00)
Patients with relapses in the 2 years before SPMS, *n* (%)	2976 (16.38)	421 (48.61)	0 (0.00)
Patients with relapses after SPMS conversion, *n* (%)	-	380 (43.88)	395 (24.64)
Onset type			
Unifocal	15,182 (87.45)	654 (77.86)	1263 (81.80)
Multifocal	2179 (12.55)	186 (22.14)	281 (18.20)
Patients with active lesions at the first visit, *n* (%)			
No	11,011 (71.99)	433 (72.29)	898 (71.90)
Yes	4285 (28.01)	166 (27.71)	351 (28.10)
Presence of MRI T2 brain lesions (RISM codification of lesion burden) at the first visit, *n* (%)			
0	515 (6.67)	15 (4.81)	34 (5.48)
1–2	808 (10.46)	15 (4.81)	41 (6.60)
3–8	3221 (41.72)	182 (58.33)	307 (49.44)
≥ 9	3177 (41.15)	100 (32.05)	239 (38.49)

*EDSS* Expanded Disability Status Scale, *MRI* magnetic resonance imaging, *RISM* Italian Multiple Sclerosis and Related Disorders Register, *SPMS*, secondary progressive multiple sclerosis

Patients defined by the HERCULES criteria were slightly older compared to ND patients (median (IQR) age: 46.60 (39.20–53.40) vs. 44.45 (37.40–51.70) years). The median (IQR) time from the first visit to SP transition was 7.08 (3.61–12.34) years in the ND cohort and 7.00 (3.50–12.15) years in the HERCULES group. The percentage of patients presenting with clinical relapses after the SP conversion differed between the two groups, with a higher percentage for the ND (43.88%) compared to the HERCULES definition (24.64%).

Table 2 displays the distribution of PIRA and RAW events in the two cohorts of SPMS patients. PIRA events were identified in 769 (88.80%) patients in the ND group and 1419 (88.52%) patients in the HERCULES-defined cohort. The median (IQR) time from the first visit to the first PIRA event was shorter in the HERCULES-criteria group compared to the ND (4.46 (1.94–9.09) vs 5.11 (2.14–9.74) years, respectively). In the HERCULES-adapted cohort, about half of PIRA events occurred before SPMS conversion (47.99%) and only a small proportion after conversion (7.61%). In comparison, ND-defined SPMS showed a higher proportion of PIRA occurring both before (56.57%) and after conversion (32.64%). These percentages are detailed in Table 2. RAW events were less frequent overall: the proportion of patients with a RAW event after SPMS conversion was higher in the ND cohort (14.08%) than in the HERCULES group (4.21%). The median time from the first visit to the first RAW event was 3.36 (1.59–7.25) years in the HERCULES group and 3.68 (1.61–7.13) years in the ND group.

**Table 2:** Assessment of PIRA and RAW events for ND and HERCULES definitions

	ND (866)	HERCULES-adapted definition (1603)
Patients presenting PIRA events, *n* (%)	769 (88.80)	1419 (88.52)
Time to PIRA, median (IQR) years	5.11 (2.14–9.74)	4.46 (1.94–9.09)
PIRA events before SPMS conversion, *n* (%)	435 (56.57)	681 (47.99)
Patients presenting PIRA events concomitant to SPMS conversion, *n* (%)	83 (10.79)	630 (44.40)
Patients presenting PIRA events after SPMS conversion, *n* (%)	251 (32.64)	108 (7.61)
Patients presenting RAW events, *n* (%)	213 (24.60)	190 (11.85)
Time to RAW, median (IQR) years	3.68 (1.61–7.13)	3.36 (1.59–7.25)
Patients presenting RAW events before SPMS conversion, *n* (%)	164 (77.00)	182 (95.79)
Patients presenting RAW events concomitant to SPMS conversion, *n* (%)	19 (8.92)	0 (0.00)
Patients presenting RAW events after SPMS conversion, *n* (%)	30 (14.08)	8 (4.21)

*PIRA* progression independent of relapse activity *RAW* relapse-associated worsening; *SPMS* secondary progressive multiple sclerosis

We evaluated the concordance between the HERCULES and the ND definition, used as the reference definition for comparison. In terms of diagnostic performance, the HERCULES definition showed a high specificity (93.4%) and a low sensitivity (37.6%), with a C-Index of 55.0%. The overall accuracy was 91.1% (90.7–91.5). The goodness of fit was supported by the non-significant Calibration slope test (*p* = 0.99), indicating overlap between observed and predicted probabilities, and by a lower AIC. A detailed summary of diagnostic performance is reported in Table 3.

**Table 3:** Diagnostic performance of HERCULES definition for nrSPMS

Neurologist’s definition as Gold Standard		HERCULES-adapted definition
True positive	Neurologist +/Algorithm +	326
False negative	Neurologist +/Algorithm–	540
False positive	Neurologist–/Algorithm +	1277
True negative	Neurologist–/Algorithm–	18,163
% true positive	%	1.6 (1.4–1.8)
% false negative	%	2.7 (2.5–2.9)
% false positive	%	6.3 (6.0–6.8)
% true negative	%	89.4 (89.0–89.8)
AIC intercept and covariate	Lower is better	4372
C-Index		0.55 (0.45–0.65)
Calibration slope p-value test	Well calibrated with* p* > 0.05	0.99
Sensitivity	% of Neurologist SP identified by the HERCULES adapted definition	37.6 (36.9–38.3)
Specificity	% of Neurologist RR identified by the HERCULES adapted definition	93.4 (93.1–93.7)
PPV	% of Neurologist SP identified by the HERCULES adapted definition already diagnosed as SP by the neurologist	20.3 (19.7–20.9)
NPV	% of Neurologist RR identified as SP by the HERCULES adapted definition	97.1 (96.9–97.3)
Accuracy		91.1 (90.7–91.5)

*PPV* positive predictive value, *NPV* negative predictive value, *AIC* Akaike Information Criterion

**Fig. 2:**
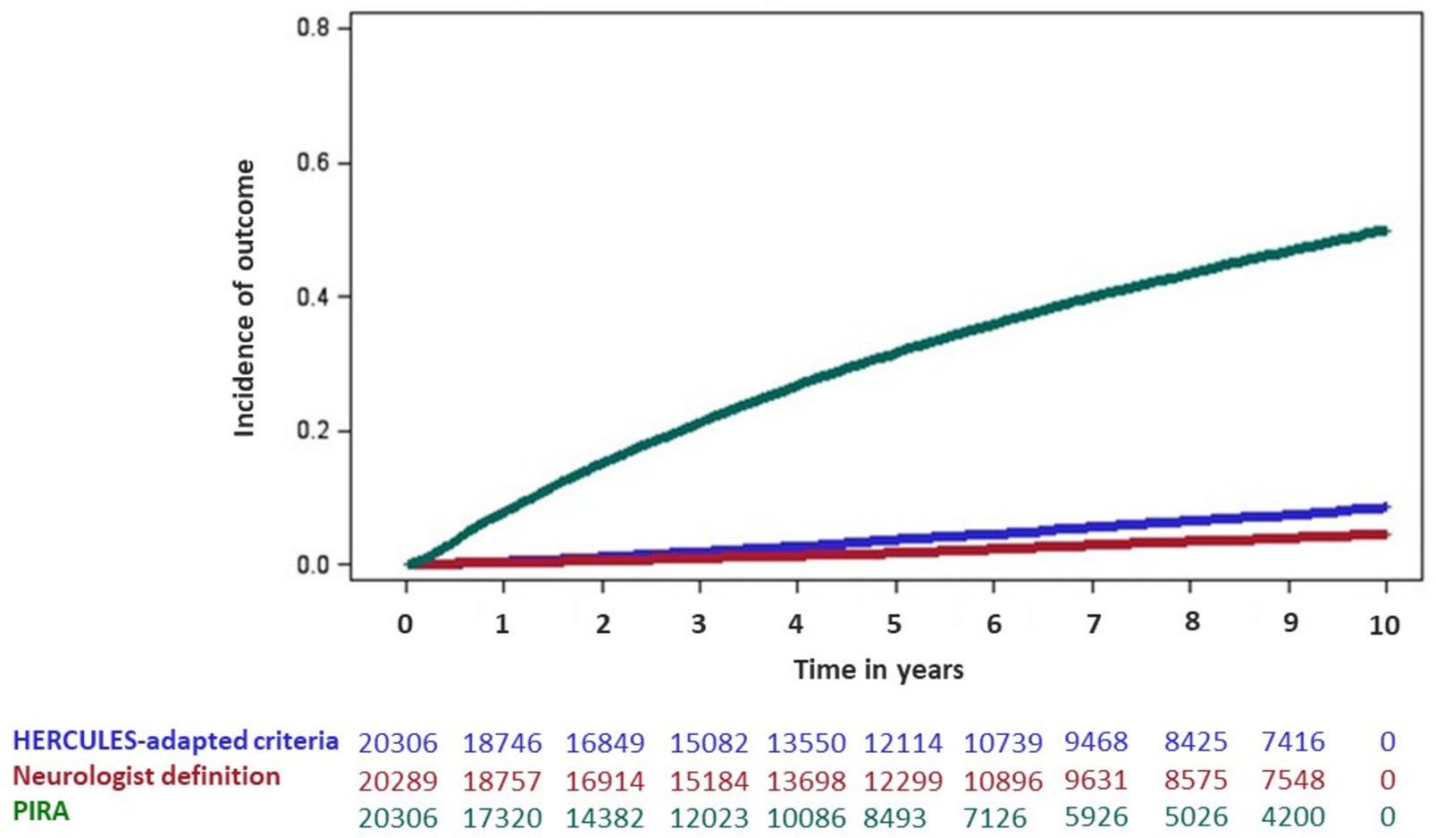
Cumulative incidence of SPMS conversion according to neurologist-defined and adapted HERCULES criteria, and cumulative incidence of PIRA over follow-up. Time is expressed in years. Numbers below the curves represent the number of subjects at risk at the corresponding time points

**Fig. 3:**
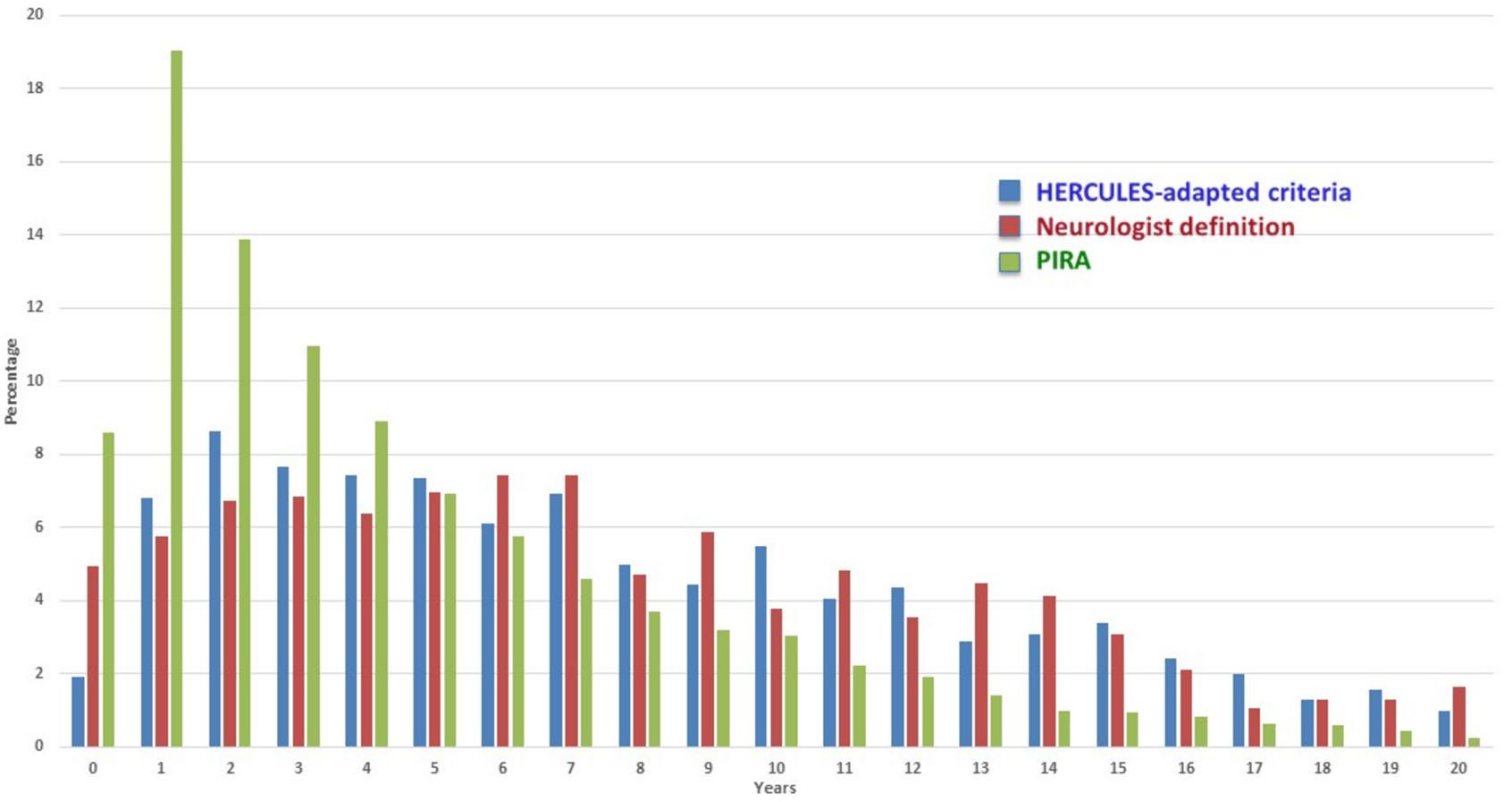
Annual distribution of time to SPMS conversion according to neurologist-defined and adapted HERCULES criteria, and time to first PIRA event. Bars represent the percentage of events occurring in each year among subjects at risk during that year

The cumulative incidence evaluation reported in Fig. 2 shows that HERCULES and ND have a very similar trend, in parallel with a higher risk of PIRA occurring since the start of the observation and persisting over time. The distribution of time to SPMS conversion according to the ND and HERCULES-adapted definitions, as well as time to the first PIRA event, is reported in Fig. 3. Results of exploratory Cox regression analyses assessing associations between baseline factors and SPMS identification according to each definition are reported in the Supplementary Material.

A subgroup analysis based on the geographical distribution of patients according to MS centers where they are followed, divided into regions of northern, central, and southern Italy, was also performed and reported in Table 4. Participants’ characteristics were similar in terms of follow-up duration, sex, presence of active lesions, symptoms at onset, age at onset, and EDSS at first evaluation. Participants followed in MS Centers in Northern Italy show a lower percentage of PIRA (35.82% vs 41.96% in the Center and 43.21% in the South) and HERCULES (5.67% vs 7.63% in the Center and 10.41% in the South).

**Table 4:** Geographical distribution of patients included in the study and outcome assessment

Variable	North	Center	South
Number of patients	8662	3615	8029
Follow-up, median (IQR) years	6.50 (3.00–11.70)	6.60 (3.00–12.20)	7.40 (3.20–13.60)
Female sex, *n* (%)	5750 (66.38)	2475 (68.46)	5385 (67.07)
Subject with presence of active lesion, *n* (%)			
No	5252 (73.53)	2320 (70.01)	4616 (71.38)
Yes	1891 (26.47)	994 (29.99)	1851 (28.62)
Presence of MRI T2 brain lesions (RISM codification of lesion burden), *n* (%)			
0	151 (4.72)	75 (6.17)	332 (8.06)
1–2	373 (11.67)	182 (14.97)	300 (7.28)
3–8	1252 (39.17)	627 (51.56)	1762 (42.76)
≥ 9	1420 (44.43)	332 (27.30)	1727 (41.91)
Type of MS onset			
Unifocal	7249 (87.83)	2993 (87.98)	6612 (85.06)
Multifocal	1004 (12.17)	409 (12.02)	1161 (14.94)
Age at onset, median (IQR)	32.80 (26.20–41.60)	32.90 (26.30–41.40)	31.10 (24.90–39.10)
Age at SPMS according to ND, median (IQR)	46.80 (39.60–53.90)	45.30 (39.90–53.60)	41.80 (35.60–49.00)
Age at SPMS according to Hercules, median (IQR)	48.50 (40.00–56.00)	46.25 (40.90–53.20)	45.80 (38.15–52.00)
Age at PIRA, median (IQR)	38.10 (30.90–46.90)	38.00 (30.70–46.50)	36.40 (29.80–44.90)
First EDSS, median (min–max)	1.50 (1.00–2.00)	1.50 (1.00–2.00)	2.00 (1.00–2.50)
SPMS patients according to ND, *n* (%)	355 (4.10)	98 (2.71)	413 (5.14)
SPMS patients according to HERCULES, *n* (%)	491 (5.67)	276 (7.63)	836 (10.41)
Patients with PIRA events, *n* (%)	3103 (35.82)	1517 (41.96)	3469 (43.21)
Patients with PIRA events before SPMS conversion according to ND, *n* (%)	2964 (95.52)	1482 (97.69)	3309 (95.39)
Patients with PIRA events concomitant with SPMS conversion according to ND, *n* (%)	32 (1.03)	8 (0.53)	43 (1.24)
Patients with PIRA events after SPMS conversion according to ND, *n* (%)	107 (3.45)	27 (1.78)	117 (3.37)
Patients with PIRA events before SPMS conversion according to HERCULES, *n* (%)	2877 (92.72)	1384 (91.23)	3090 (89.07)
Patients with PIRA events concomitant with SPMS conversion according to HERCULES, *n* (%)	198 (6.38)	106 (6.99)	326 (9.40)
Patients with PIRA events after SPMS conversion according to HERCULES, *n* (%)	28 (0.90)	27 (1.78)	53 (1.53)
Time to SPMS according to ND, median (IQR) years	6.24 (2.87–11.23)	6.40 (2.97–11.91)	7.01 (3.10–12.91)
Time to SPMS according to HERCULES, median (IQR) years	6.21 (2.86–11.27)	6.17 (2.92–11.43)	6.80 (2.98–12.59)
Time to PIRA, median (IQR) years	4.03 (1.72–8.04)	3.62 (1.59–7.24)	4.03 (1.68–8.11)
Patients with relapses in 2 years before SPMS according to ND, *n* (%)	143 (40.28)	46 (46.94)	232 (56.17)
Patients with relapses in 2 years before SPMS according to HERCULES, *n* (%)	0 (0.00)	0 (0.00)	0 (0.00)
Patients with relapses after SPMS according to ND, *n* (%)	137 (38.59)	50 (51.02)	193 (46.73)
Patients with relapses after SPMS according to HERCULES, *n* (%)	112 (22.81)	66 (23.91)	217 (25.96)

*EDSS* Expanded Disability Status Scale, *MRI* magnetic resonance imaging *ND* neurologist definition, *PIRA* progression independent of relapse activity, *RAW* relapse-associated worsening, *RISM* Italian Multiple Sclerosis and Related Disorders Register, *SPMS* secondary progressive multiple sclerosis

## Discussion

The identification of SPMS transition remains a crucial clinical challenge since the absence of a widely recognized definition in differentiating relapsing and progressive forms. Nowadays, neurologists diagnose SPMS retroactively considering a history of progressive impairment following an initial relapsing phase. [17, 18] The difficulty of detecting SPMS patients also seems to affect whether they will be characterized as active or nonrelapsing [8].

In addition, using adapted HERCULES criteria, in this multicenter retrospective study we identified a distinct nrSPMS phenotype characterized by older age and fewer relapses after conversion. Only a small proportion of post-conversion relapses resulted in RAW, supporting the concept that disability accumulation in this group is largely driven by PIRA mechanisms. These findings are consistent with prior studies reporting a predominance of nonactive SPMS and highlighting PIRA as the main driver of disability. A recent study [8] identified SPMS patterns in the cohort of the Danish MS Register, outlining a higher percentage of nonrelapsing SPMS patients at the time of conversion and a higher cumulative probability of being nonactive by the end of follow-up. The different criteria applied to the RISM cohort could explain the discrepancy with this study. The population of nonactive SPMS in the RISM database has been also recently described [19]: compared to active SPMS, the patients in this cohort were older and had a longer disease duration.

Considering the results of the study of Chisari et al. [19], the proportion of patients presenting PIRA at 24 months follow-up was comparable in the active and nonactive groups, confirming that PIRA is largely responsible for progression [2, 4, 20, 22].

A recent study by Portaccio et al. [22] highlighted that a single PIRA event, particularly in the case of sustained PIRA, is sufficient to start progression in most of the patients. In addition, in our cohort, PIRA events were more frequent than RAW and occurred earlier than formal SPMS conversion, reinforcing the notion that PIRA may precede and signal the transition to secondary progression. Time to first PIRA was shorter than time to SPMS diagnosis, and accounting for PIRA reduced the association between different SPMS definitions, suggesting that early PIRA substantially influences disease course classification. These observations support the view that the occurrence of PIRA should prompt closer monitoring for progression and potential treatment reassessment.

Diagnostic performances of standardized definitions for the diagnosis of SPMS are the subject of compelling and recent debate. A recent study analyzed data from five European MS registries and showed different proportions of identified SPMS patients employing three objective classification methods, the MSBase algorithm [23], the EXPAND criteria, and a decision tree-based algorithm [24]. The sensitivity differed between the methods (47.4% EXPAND, 75.7% MSBase algorithm, 83.5% decision tree), while the specificity resulted similar. The overall accuracy of each method compared to the clinical SPMS assignment was 79.1% for EXPAND, 85.4% for the MSBase algorithm, and 83.7% for the decision tree classifier [24]. Braune et al. outlined that the specificity of the MSBase criteria for SPMS was also high (89.6% in the group of patients diagnosed with SPMS and 96.2% including RRMS patients without transition), in parallel with low values of sensitivity (32%) and accuracy (61.4%) [25]. The HERCULES criteria, compared to the ND, applied to our cohort resulted in low sensitivity (37.6%) and high levels of specificity (93.4%), in line with previous results. Nonetheless, it is important to emphasize a high level of accuracy of 91.1% of HERCULES-adapted criteria. Considered together, these performances indicate that the HERCULES-adapted criteria offer a highly specific and therefore useful approach to identify well-defined SPMS cases with high confidence. The low sensitivity should be viewed in the context of the reference standard, as clinician-defined SPMS is often heterogeneous and delayed. These measures were not intended to support individual-level risk prediction, but to quantify overall agreement and classification performance in the absence of a true gold standard for SPMS diagnosis.

The observed differences between ND and HERCULES-defined SPMS populations should be interpreted considering the intrinsic differences between the two diagnostic frameworks. By design, these definitions capture inherently different patient populations. This fundamental distinction could explain several of the observed findings, including younger age and higher inflammatory activity in ND-defined SPMS. Conversely, the lower frequency of PIRA observed in ND-defined SPMS may reflect reduced exposure time for relapse-independent progression, as disability changes occurring in temporal proximity to relapses cannot be classified as PIRA. In this context, the higher number of SPMS diagnoses and the earlier timing of SPMS identification observed with HERCULES criteria are particularly noteworthy because these findings suggest that criteria-based definitions may reclassify early PIRA-related disability accumulation as SPMS in the contemporary treatment era. This concept aligns with the ongoing debate surrounding the concept of how specific PIRA truly is for inflammation-independent progression. Emerging evidence supports a shift from purely clinical definitions of PIRA toward a biologically driven framework grounded in MS pathogenic mechanisms and MRI correlates, moving beyond traditional clinical descriptors [30].

We also performed an analysis of geographical discrepancies in SPMS definition. Patients followed in MS Centres in Northern Italy show a lower percentage of PIRA and HERCULES defined patients, compared to MS Centres of Central and Southern Italy. A previous work of the RISM assessed geographical socio-economic factors and characteristics of local MS centers in Italy, underscoring their role of primary factor influencing phenotype at initial neurological evaluation [26]. Further research will be useful to explore possible determinants and consequences of regional variability.

Some key limitations of the study must be acknowledged. A primary limitation is that the algorithm depends solely on EDSS records, with all the related limits of this scale. [27] Groups differed in disability severity, with EDSS < 3.0 in the ND reference group and EDSS 3.0–6.5 in the HERCULES definition. Observed discrimination may partly reflect disability level rather than disease course, potentially leading to an overestimation of the ability to identify SPMS. Furthermore, as discussed, the stringent criteria of the data-driven definition adopted in our analysis may explain the different proportion of nrSPMS patients reported in other studies. [8] Potential cohort-related biases should be considered when interpreting these findings. Specifically, the lower SPMS diagnostic rates observed in our cohort compared with classic natural history studies, in which approximately 23–25% of patients converted to SPMS over 10–11 years, likely reflect differences in follow-up, clinical characteristics and study design [31, 32].

Consistent with other retrospective studies based on disease registries, data incompleteness and entry inaccuracies cannot be excluded. A major limitation of the adapted HERCULES definition is the exclusion of radiological activity, as MRI data are not standardized across the RISM network, resulting in a classification based solely on clinical parameters. This distinction is clinically relevant and should be acknowledged when interpreting our findings. Despite using the ND as the reference definition for comparison, we cannot rule out the possibility that the neurologists treating patients included in our cohort underdiagnosed SPMS because of a diagnostic delay that may have been caused by the hesitancy to diagnose secondary progression [28] and the later onset of the progressive phase [29]. Furthermore, in this study we did not account for DMT class or treatment patterns in our cohort and relapse suppression from high-efficacy therapies may have influenced nrSPMS classification, representing a potential confounder to consider when interpreting these results. No formal sample size calculation was performed, as this was a retrospective, exploratory, registry-based study.

Our findings underline the importance of using algorithms for SPMS diagnosis, particularly when they are applied as standardized end points in epidemiological research based on MS registry data. In addition, the study is based on the RISM patient population, which is highly representative of the Italian MS patient population. [16] With participants from 125 MS centers (about 81% of all centers of RISM network), the population of RISM included in this study is also highly representative, being geographically widely distributed.

In conclusion, applying nrSPMS criteria adapted from the HERCULES trial to our cohort reliably identified a distinct nrSPMS subgroup, accounting for 7.89% of our study population. NrSPMS patients were less likely to experience relapses after SP conversion and had even fewer RAW events, while PIRA emerged as the predominant CDA event, being markedly more frequent than RAW and occurring earlier in the HERCULES group than in the ND. Overall, these findings reinforce the need to prevent disability accumulation primarily driven by smoldering neuroinflammation.

## Supplementary Information

Below is the link to the electronic supplementary material.

Supplementary file1 (DOCX 17 KB)

## Data Availability

Anonymized data will be shared on reasonable request from a qualified investigator.
