# Supplementary material for: HerMeS: a registry-based evaluation of the HERCULES criteria for identifying nonrelapsing SPMS
Source: J Neurol. 2026 Mar 9;273(3):193. doi: 10.1007/s00415-026-13716-1 (PMC12971826; doi:10.1007/s00415-026-13716-1)
Supplement: Supplementary file 1 — Supplementary file1 (DOCX 17 KB) [file 415_2026_13716_MOESM1_ESM.docx]

|  |  | **ND** | | **HERCULES** | |
| --- | --- | --- | --- | --- | --- |
| **Parameter** | **Category** | **HR (95% CI)** | **p-value** | **HR (95% CI)** | **p-value** |
| Gender | Male vs Female | **1.34 ( 1.17-1.54)** | **<.0001** | **1.22 ( 1.10-1.35)** | **0.0002** |
| Active lesions at the first visit | Yes vs No | 0.95 ( 0.79-1.15) | 0.6159 | 1.03 ( 0.91-1.17) | 0.6746 |
| MRI T2 brain lesions at first visit | 1-2 vs 0 | 0.64 ( 0.31-1.31) | 0.2216 | 0.76 ( 0.48-1.20) | 0.2384 |
|  | 3-8 vs 0 | 1.22 ( 0.72-2.08) | 0.4633 | 0.95 ( 0.66-1.35) | 0.762 |
|  | >=9 vs 0 | 1.10 ( 0.64-1.90) | 0.7301 | 1.26 ( 0.87-1.81) | 0.2159 |
| Type of clinical onset | Multifocal vs Monofocal | **1.51 ( 1.28-1.79)** | **<.0001** | **1.26 ( 1.10-1.44)** | **0.0006** |
| Number of relapses in 2 years before firs visits | 1 vs 0 | 1.11 ( 0.94-1.32) | 0.2328 | **0.71 ( 0.62-0.81)** | **<.0001** |
|  | >=2 vs 0 | 0.89 ( 0.65-1.21) | 0.4522 | **0.61 ( 0.48-0.77)** | **<.0001** |
| Oligoclonal banding | Positive vs Negative | **1.47 ( 1.12-1.92)** | **0.005** | 1.18 ( 0.98-1.43) | 0.0815 |
| Age at onset (years) |  | **1.04 ( 1.03-1.05)** | **<.0001** | **1.05 ( 1.05-1.06)** | **<.0001** |
| First EDSS |  | **1.37 ( 1.31-1.42)** | **<.0001** | **1.31 ( 1.27-1.35)** | **<.0001** |
| Time to first visit (months) |  | **1.02 ( 1.00-1.04)** | **0.0476** | **1.06 ( 1.05-1.08)** | **<.0001** |

**Supplementary Table 1.** Factors associated with SPMS conversion according to neurologist-driven and HERCULES definitions: Cox proportional hazards analyses.
